# HPTLC-DESI-HRMS-Based Profiling of Anthraquinones in Complex Mixtures—A Proof-of-Concept Study Using Crude Extracts of Chilean Mushrooms

**DOI:** 10.3390/foods9020156

**Published:** 2020-02-06

**Authors:** Annegret Laub, Ann-Katrin Sendatzki, Götz Palfner, Ludger A. Wessjohann, Jürgen Schmidt, Norbert Arnold

**Affiliations:** 1 Department of Bioorganic Chemistry, Leibniz Institute of Plant Biochemistry, Weinberg 3, D-06120 Halle (Saale), Germany; alaub@ipb-halle.de (A.L.); asend@web.de (A.-K.S.); wessjohann@ipb-halle.de (L.A.W.); jschmidt@ipb-halle.de (J.S.); 2 Departamento de Botanica, Facultad de Ciencias Naturales y Oceanograficas, Universidad de Concepcion, Casilla 160-C, Concepcion, Chile; gpalfner@udec.cl

**Keywords:** HPTLC-MS coupling, HPTLC, negative ion DESI-HR-MS/MS, anthraquinones, Chilean mushrooms, genus *Cortinarius*

## Abstract

High-performance thin-layer chromatography (HPTLC) coupled with negative ion desorption electrospray ionization high-resolution mass spectrometry (DESI-HRMS) was used for the analysis of anthraquinones in complex crude extracts of Chilean dermocyboid Cortinarii. For this proof-of-concept study, the known anthraquinones emodin, physcion, endocrocin, dermolutein, hypericin, and skyrin were identified by their elemental composition. HRMS also allowed the differentiation of the investigated anthraquinones from accompanying compounds with the same nominal mass in the crude extracts. An investigation of the characteristic fragmentation pattern of skyrin in comparison with a reference compound showed, exemplarily, the feasibility of the method for the determination of these coloring, bioactive and chemotaxonomically important marker compounds. Accordingly, we demonstrate that the coupling of HPTLC with DESI-HRMS represents an advanced and efficient technique for the detection of anthraquinones in complex matrices. This analytical approach may be applied in the field of anthraquinone-containing food and plants such as *Rheum* spp. (rhubarb), *Aloe* spp., *Morinda* spp., *Cassia* spp. and others. Furthermore, the described method can be suitable for the analysis of anthraquinone-based colorants and dyes, which are used in the food, cosmetic, and pharmaceutical industry.

## 1. Introduction

Anthraquinones represent a large family of naturally occurring pigments, which are produced by plants, microbes, lichens, insects, and fungi [1]. Besides their coloring properties, these natural products exhibit a broad range of bioactivities such as antibacterial, antiparasitic, anti-inflammatory, fungicidal, insecticidal, laxative, antiviral, and anticancer but also DNA intercalating properties [2,3,4,5,6,7]. The chemical structure of anthraquinones is based on an anthracene skeleton with two keto groups in position 9 and 10. The basic core unit can be further substituted at various positions and connected with sugar molecules, forming the corresponding glycosides [8,9].

In the literature, about 700 anthraquinone derivatives are described, in which emodin, physcion, catenarin, and rhein are the most frequently reported [9,10,11]. Two hundred of these are described for flowering plants, which also occur in edible plants and vegetables such as *Rheum*, *Aloe* and *Cassia* species, while the remaining ones are produced by lichens and fungi [7,8,12].

The genus *Cortinarius* (including Dermocybe) is one of the most diverse genera of basidiomycetous fungi containing a great variety of anthraquinones [13,14,15]. The occurrence and distribution of these pigments is closely linked to species diversity and allows their use as chemotaxonomic marker compounds in species delimination [16,17,18,19,20,21,22].

The analysis of anthraquinones is of interest due to their wide range of application. A continuous improvement of the analytical techniques is needed to overcome difficulties with respect to interference with various types of matrices and low abundance of the analytes within complex mixtures [7].

Thin-layer chromatography is an effective method for the chromatographic separation of anthraquinones [23,24,25,26]. Furthermore, several mass spectrometry-based methods have been developed for a deep analysis of anthraquinones providing characteristic [M-H]^−^ ions in negative ion mode [27,28,29].

Desorption electrospray ionization mass spectrometry (DESI-MS) represents a powerful ambient ionization mass spectrometric technique, which enables a direct ionization of analytes from surfaces with subsequent mass spectrometric detection [30,31,32]. The coupling of DESI-MS with high-performance thin-layer chromatography (HPTLC) provides a robust methodological approach for the separation and highly sensitive detection of secondary metabolites in plants and fungi [33,34,35]. Furthermore, this method is suitable for the fingerprint analysis of crude extracts in natural product research [36,37]. Recently, the detection of excreted polyhydroxyanthraquinones from the surface of fungal culture agar plates using DESI-MS in negative ion mode was reported [38].

In the present paper, we report the development of a rapid profiling method of anthraquinones, exemplified with the analysis of different crude extracts from Chilean dermocyboid Cortinarii concerning their anthraquinone pattern based on the combination of HPTLC with negative ion DESI-HRMS. For this proof-of-concept study, extracts from fruiting bodies of six dermocyboid Cortinarii were investigated for the occurrence of the known anthraquinones emodin, physcion, endocrocin, dermolutein, hypericin, and skyrin. Furthermore, the possibility of performing MS/MS experiments on the desorbed analytes directly from the HPTLC plate was exemplarily shown for the bisanthraquinone skyrin in comparison with data obtained from direct-infusion MS experiments.

## 2. Materials and Methods

### 2.1. Reagents and Chemicals

The authentic reference compounds endocrocin (**3**), hypericin (**5**) and skyrin (**6**) were available from the in-house compound library of the Department of Bioorganic Chemistry, Leibniz Institute of Plant Biochemistry (IPB), Halle (Saale), Germany. Methanol and toluene were used at analytical grade. Ethyl formate was purchased from Merck (Darmstadt, Germany) and formic acid from Roth (Karlsruhe, Germany). LC-MS grade methanol was obtained from Merck (Darmstadt, Germany), and purified water was prepared by Merck Millipore Milli-Q equipment (Darmstadt, Germany).

### 2.2. Sampling Sites and Extraction

Fruiting bodies of *C.* (D.) *austronanceiensis*, *C.* (D.) *icterina*, *C.* (D.) *icterinula*, *C.* (D.) *obscuro-olivea*, *C.* (D.) spec., and *C.* (D.) *viridulifolius* were collected in Chile (detailed information see Table S1). Voucher specimens are deposited in the Fungarium of Concepción University (CONC-F). A duplicate is deposited at the Leibniz Institute of Plant Biochemistry.

Air-dried fruiting bodies (2 g) were homogenized using 15 mL of acetone in a blender followed by an ultrasonic extraction for 15 min to remove interfering compounds such as fatty acids from the material. After vacuum-supported filtration, the fungal material residue was further extracted twice with 15 mL methanol each. The resulting extracts were filtrated and dried under reduced pressure using a rotary evaporator. The crude methanolic extracts were redissolved in methanol and directly spotted on the HPTLC plate for chromatographic separation.

### 2.3. HPTLC

HPTLC was performed on Glass HPTLC Silica gel 60 F_254_ plates (10 × 10 cm, layer thickness 150–200 µm, Merck) using a mixture of toluene, ethyl formate, and formic acid (10:5:3; *v*/*v*/*v*) as a mobile phase. After air drying, the developed plates were parted by a glass cutter and subjected to the mass spectral analysis. For documentation and Rf-value determination, a CAMAG TLC visualizer (CAMAG, Muttenz, Switzerland) was used with the software winCATS (version 1.4.9.2001, CAMAG, Switzerland).

### 2.4. DESI-Orbitrap-MS and MS^2^

All experiments were performed using a 2D-DESI source (Omnispray System OS-3201, Prosolia, Indianapolis, IN, USA) coupled to an Orbitrap Elite mass spectrometer (Thermo Fisher Scientific, Bremen, Germany) operated in the negative ion mode. The DESI source settings were as follows: spray voltage, 3 kV; solvent flow rate, 2 µL/min; nebulizing gas (nitrogen), pressure, 7 bar; tip-to-surface distance, 2–2.5 mm; tip-to-inlet distance, 3.5 mm; incident angle (relative to the surface plane), 55°. The DESI spray solvent was 50:50 (*v*/*v*) methanol/water. MS experiments were performed by continuously scanning every HPTLC band in the y-direction (from Rf 0 to 1.0) at a surface velocity of 200 µm/s while acquiring mass spectra in full scan mode (*m*/*z* 150–1500; resolution 30,000) and 150 µm/s in MS^2^ mode. Collision-induced dissociation was performed using normalized collision energies (NCE) of 35 and 50 (arbitrary units) and an isolation width of ± 2 Da. The data were evaluated using the software Xcalibur 2.2 SP1 (Thermo Fisher Scientific).

## 3. Results and Discussion

### 3.1. Method Development

**Figure 1:**
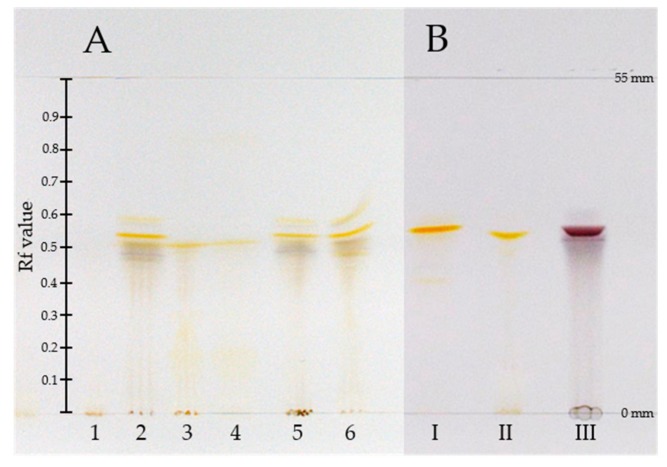
High-performance thin-layer chromatograph (HPTLC) of (**A**) methanolic crude extracts of *C.* (D.) spec. (1), *C.* (D.) *austronanceiensis* (2), *C.* (D.) *icterina* (3), *C.* (D.) *icterinula* (4), *C. obscuro-olivea* (5), *C. viridulifolius* (6) and (**B**) reference compound skyrin (**6**, I), endocrocin (**3**, II), and hypericin (**5**, III) (mobile phase: toluene, ethyl formate, and formic acid (10:5:3; *v*/*v*/*v*) distance from application line to solvent front: 55 mm).

The normal-phase HPTLC plates were developed for 55 mm in one dimension to enable the separation of anthraquinones according to their polarity (Figure 1). The geometry of the source, the composition of the spray solvent, the flow rate as well as the scanning rate were optimized for the analysis. To enhance the ionization and desorption efficiency, different mixtures of methanol and water (with and without formic acid as additive) were tested as spray solvents. A mixture of methanol and water of 1:1 (*v*/*v*) yielded the best results. During optimization, a flow rate of 2 µL/min showed good results to obtain adequate signal intensities. On the other hand, higher flow rates led to a partial detachment of silica gel particles. Additionally, different velocities of the DESI spray head were tested to obtain an efficient number of precursor ions for the MS^2^ experiments. Lower scan rates led to better signal intensities due to the better desorption of the analytes from the surface of the HPTLC plates. Therefore, we used a lower velocity for the MS^2^ experiments in the final measurements than in the full scan runs. Each band was recorded by scanning the surface in the y-direction (Rf 0 to 1.0) with an automated DESI source coupled to an Orbitrap Elite mass spectrometer within a total run time of 4.6 min. Before starting the experiment, the spray head was positioned on the application line of the HPTLC followed by the manual start of the MS measurement.

### 3.2. Profiling of Anthraquinones in Crude Extracts

**Figure 2:**
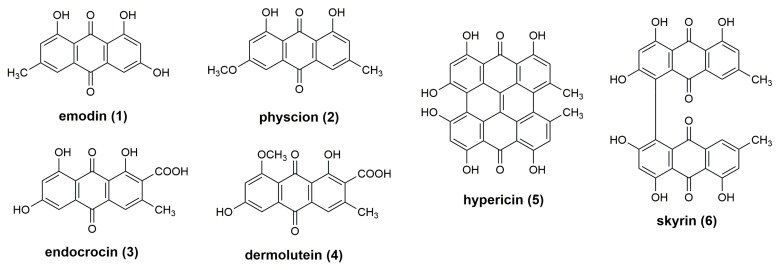
Structures of investigated anthraquinones **1–6**.

The pigment pattern of the methanolic extracts of dermocyboid Cortinarii *Cortinarius* (Dermocybe) *austronanceiensis*, *C*. (D.) *icterina*, *C*. (D.) *icterinula*, *C*. (D.) *obscuro-olivea*, *C*. (D.) spec., and *C*. (D.) *viridulifolius* (Figure 1) was analyzed by high-performance thin-layer chromatography (HPTLC) coupled to desorption electrospray ionization (DESI) mass spectrometry in the negative ion mode. An unspotted HPTLC band was scanned to assign background related peaks (Figure S1) and to ensure the absence of the target compounds before applying the crude extracts on the HPTLC plate. No anthraquinone-related peaks could be detected by scanning the empty band on the HPTLC plate after running with the solvent system. This is demonstrated by the extracted ion chromatograms based on the theoretical calculated *m*/*z* value of the [M-H]^−^ ions using an 25 ppm window (four decimals) (Figure S2).The established analytical method was applied to identify anthraquinones 1–6 (Figure 2). These anthraquinones were chosen for this proof-of-concept study because their occurrence in different *Cortinarius* and *Dermoybe* species is described in the literature [15]. The assignment of the structures is based on their elemental composition determined by high-resolution mass spectrometry (HRMS) (Table 1 and Table S2).

**Figure 3:**
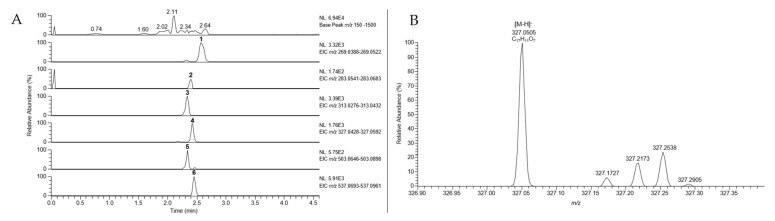
(**A**) Extracted ion chromatograms (EIC, mass window: 25 ppm) of anthraquinones 1-6 from crude extract of *Cortinarius* (D.) *austronanceiensis*, (**B**) Extracted ion chromatogram (EIC) of dermolutein (3, *m*/*z* 327) acquired during DESI-HR-MS measurement of methanolic extract from *C.* (*D*.) *austronanceiensis*.

The negative ion DESI mass spectra of the methanolic extract of *C*. (D.) *austronanceiensis* afforded characteristic deprotonated ions of emodin (1, [M-H]^−^ at *m*/*z* 269.0450 calcd for C_15_H_9_O_5_^−^ 269.0455), physcion (2, [M-H]^−^ at *m*/*z* 283.0611 calcd for C_16_H_11_O_5_^−^ 283.0612), endocrocin (3, [M-H]^−^ at *m*/*z* 313.0349 calcd for C_16_H_9_O_7_^−^ 313.0354), dermolutein (4, [M-H]^−^ at *m*/*z* 327.0505 calcd for C_17_H_11_O_7_^−^ 327.0510), hypericin (5, [M-H]^−^ at *m*/*z* 503.0763 calcd for C_30_H_15_O_8_^−^ 503.0772) and skyrin (6, [M-H]^−^ at *m*/*z* 537.0817 calcd for C_30_H_17_O_10_^−^ 537.0827) (Figure 3A). For the data evaluation, the target *m*/*z* values were extracted from the total ion chromatogram using a 25 ppm mass window with a mass accuracy of four decimals to obtain the corresponding extracted ion chromatograms (EICs) for each analyte. The EICs for the methanolic extracts of *C*. (D.) *icterina*, *C*. (D.) *icterinula*, *C*. (D.) *obscuro-olivea*, *C*. (D.) spec., and *C*. (D.) *viridulifolius* are shown in Figures S3–S7, and the presences of the different target analytes within the extracts are visualized in Table 1. Due to the resolving power of the orbitrap detector, a differentiation of isobaric ions was possible as shown in the EIC of dermolutein (4, Figure 3B). The anthraquinone peak *m*/*z* 327.0505 is clearly separated from other accompanying ions at the same nominal mass using a resolution of 30,000.

Comparing the pigment patterns of the different fungal extracts (Table 1), all targets could be detected in the methanolic extracts of *Cortinarius* (D.) *austronanceiensis*, *C.* (D.) *obscuro-olivea* and *C*. (D.) *viridulifolius*. The naphthodianthrone hypericin (5) and the bisanthraquinone skyrin (6) were not detectable along the HPTLC bands of *C*. (D.) *icterina*, *C*. (D.) *icterinula* and *C*. (D.) spec.

Based on the retention time and the velocity of scanning the HPTLC bands (see Equation (1)), Rf values can be calculated and compared with the Rf values determined directly from the HPTLC plate (Table 2). The results of the developed HPTLC plates of the extracts and the reference compounds (see Figures S7–S10) were reproducible and comparable, exemplified based on the extracted ion chromatograms of endocrocin (Figure S11). Therefore, the determination of Rf values based on the retention time of the HPTLC-DESI-MS measurements of UV/VIS inactive analytes could be possible.

Rf = t_R_ (min) × velocity (mm/s) × 60 × 1/distance from application line to solvent front (mm) Rf = t_R_ × 0.200 mm/s × 60 × 1/55 mm (1)

### 3.3. Structural Characterization Using MS^2^ Experiments

**Figure 4:**
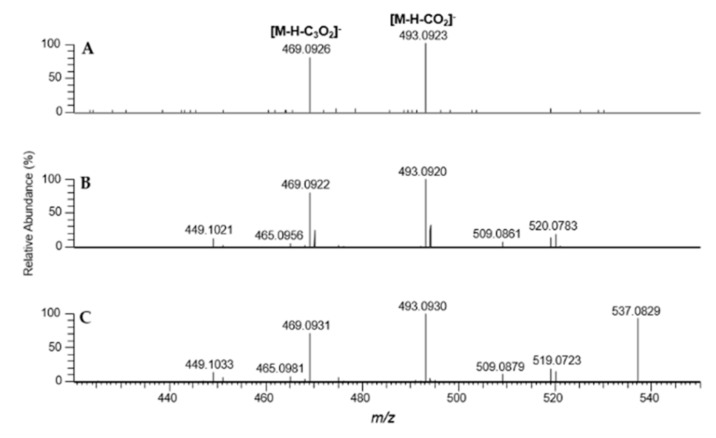
HPTLC-DESI-HR-MS^2^ data of skyrin (6); (**A**) from fungal extract of *Cortinarius* (D.) *austronanceiensis* (NCE: 50%); (**B**) skyrin standard (NCE 35%); (**C**) direct infusion ESI-HR-MS^2^ (NCE 30%).

As an example, the fragmentation behavior of the bisanthraquinone skyrin (6) was investigated by a MS^2^ measurement compared with the results obtained directly from the extract, data of a reference compound measured by HPTLC-DESI-HRMS and with direct infusion DESI-HRMS (Figure 4A–C, Table S3). Skyrin (6), in its MS^2^ spectrum, shows a base peak ion at *m*/*z* 493.0923 ([M-H-CO_2_]^−^, calcd for C_29_H_17_O_8_^−^ 493.0929, Figure 4A, Table S3). Furthermore, a loss of carbon suboxide (C_3_O_2_) is observed at *m*/*z* 469.0926 (calcd for C_27_H_17_O_8_^−^ 469.0929), indicating a 1,3-dihydroxybenzene feature, which is also described for flavones and other polyphenols [39,40]. The obtained data are in good agreement with the reported MS^2^ data of skyrin [41].

## 4. Conclusions

Crude extracts of six Chilean dermocyboid Cortinarii were investigated by HPTLC-negative ion DESI-HRMS concerning the occurrence of the anthraquinones physcion (1), emodin (2), endocrocin (3), dermolutein (4), hypericin (5), and skyrin (6). The compounds were identified by their elemental composition. It should be pointed out that the high-resolution mass spectrometry (HRMS) approach also allows a mass spectral distinction of isobaric ions as demonstrated for the detection of dermolutein (4) whose nominal mass is accompanied by other compounds in the crude extract. Furthermore, the implementation of fragmentation experiments (MS^2^) for anthraquinones on HPTLC surfaces is possible, as exemplarily shown for the detection of skyrin (6) in the extract of *C.* (D) *austronanceiensis*, and could be a valuable tool for the presence of these compound classes. The corresponding results are in good agreement with the data obtained by direct infusion and in comparison with the LC-MS data reported in literature.

HPTLC provides good separation efficiencies and can be performed in an automated and controlled way with respect to the sample application and the development of the plate. In classical approaches, a derivatization of the HPTLC plate is needed; however, combined with DESI-MS, this step is not required. Although the separation power of HPTLC is lower than in (U)HPLC, several analyses can performed with one plate and within a short analysis time. In case of the presented approach a HPTLC plate (total length 100 mm) with a developing length of 55 mm, and a total scanning time of only 4.6 min was necessary to obtain the presented results. After the extraction of the material, no further sample preparation steps are necessary, and the crude extracts can be directly applied to the plate, representing an advantage compared with other analytical techniques.

In summary, the obtained results illustrate the feasibility and capacity of HPTLC-DESI-HRMS to provide a rapid first screening method for the analysis of anthraquinones in complex mixtures, which may be used in the analysis of anthraquinones in food, plants, fungi, dyes, and cosmetic and pharmaceutical products.

## Figures and Tables

**Table 1:** Detected anthraquinones (**1**–**6**) using HPTLC-desorption electrospray ionization (DESI)-high-resolution mass spectroscopy (HRMS).

No.	Elemental Composition [M-H]^−^	Theoretical *m*/*z* [M-H]^−^	*C.* (D.) *austronanceiensis*	*C.* (D.) *icterina*	*C.* (D.) *icterinula*	*C.* (D.) *obscuro-olivea*	*C.* (D.) spec.	*C.* (D.) *viridulifolius*
**1**	C_15_H_9_O_5_^−^	269.0455	+	+	+	+	+	+
**2**	C_16_H_11_O_5_^−^	283.0612	+	+	n.d.	+	+	+
**3**	C_16_H_9_O_7_^−^	313.0354	+	+	+	+	n.d.	+
**4**	C_17_H_11_O_7_^−^	327.0510	+	+	+	+	+	+
**5**	C_30_H_15_O_8_^−^	503.0772	+	n.d.	n.d.	+	n.d.	+
**6**	C_30_H_17_O_10_^−^	537.0827	+	n.d.	n.d.	+	n.d.	+

n.d. = not detected; + = detected.

**Table 2:** Rf value and calculation from crude extract of *C*. (D.) *austronanciensis* (Figure 1, band 2).

Compound	Rf (experimental)	t_R_ DESI (min)	Rf (calculated)	Spot Color Visible Light	Spot Color UV Light (254 nm)	Spot Color UV Light (366 nm)
**1**	0.58	2.57	0.56	yellow	dark	orange
**2**	0.54	2.40	0.52	yellow	dark	orange
3 *	0.5	2.34	0.51	yellow	dark	orange
**4**	0.49	2.42	0.53	yellow	dark	red
5 *	0.5	2.34	0.51	black	dark	red
6 *	0.53	2.45	0.53	yellow-orange	dark	red brown

* Confirmed with reference compound.

## Supplementary Materials

The following are available online at https://www.mdpi.com/2304-8158/9/2/156/s1, Figure S1: (A) Total ion chromatogram of an empty HPTLC band after development with an eluent system (toluene, ethyl formate, and formic acid (10:5:3; *v*/*v*/*v*)), (B) Corresponding full MS spectrum to A (averaged over Rt 0–4.6 min) showing background related peaks. Figure S2: Total ion (A), base peak (B) and extracted ion chromatograms of an unspotted HPTLC band showing no anthraquinone related peaks (C–H)., Figure S3: Base peak chromatogram (A) and extracted ion chromatograms (EICs, B–G) based on the theoretical masses of the investigated anthraquinones (1–6) obtained from the methanolic crude extract of *Cortinarius* (D.) *icterina.*, Figure S4: Base peak chromatogram (A) and extracted ion chromatograms (EICs, B–G) based on the theoretical masses of the investigated anthraquinones (1–6) obtained from the methanolic crude extract of *Cortinarius* (D.) *icterinula.*, Figure S5: Base peak chromatogram (A) and extracted ion chromatograms (EICs, B–G) based on the theoretical masses of the investigated anthraquinones (1–6) obtained from the methanolic crude extract of *Cortinarius* (D.) *obscuro*-*olivea.*, Figure S6: Base peak chromatogram (A) and extracted ion chromatograms (EICs, B–G) based on the theoretical masses of the investigated anthraquinones (1–6) obtained from the methanolic crude extract of *Cortinarius* (D.) spec., Figure S7: Base peak chromatogram (A) and extracted ion chromatograms (EICs, B–G) based on the theoretical masses of the investigated anthraquinones (1–6) obtained from the methanolic crude extract of of *Cortinarius* (D.) *viridulifolius.*, Figure S8: Total ion and extracted ion chromatogram (EIC) of reference compound skyrin (6) and the corresponding HRMS spectrum., Figure S9: Total ion and extracted ion chromatogram EIC) of reference compound endocrocin (3) and the corresponding HRMS spectrum., Figure S10: Total ion and extracted ion chromatogram (EIC) of reference compound hypericin (5) and the corresponding HRMS spectrum., Figure S11: Comparison of extracted ion chromatograms (EICs) of endocrocin (3, *m*/*z* 313) acquired during DESI-HR-MS measurement of methanolic extract from *C.* (*D*.) species and the reference compound., Table S1: Origin of fungal material. Table S2: Detected anthraquinones (1–6), their elemental composition and exact masses., Table S3: Key ions in the negative ion ESI-MS^n^ spectra of skyrin (6). The spectroscopic data are available at RADAR [42].
