# Supplementary material for: HPTLC-DESI-HRMS-Based Profiling of Anthraquinones in Complex Mixtures—A Proof-of-Concept Study Using Crude Extracts of Chilean Mushrooms
Source: Foods. 2020 Feb 6;9(2):156. doi: 10.3390/foods9020156 (PMC7074031; doi:10.3390/foods9020156)
Supplement: Supplementary file 1 [file foods-09-00156-s001.pdf]

HPTLC-DESI-HRMS based Profiling of Anthraquinones in Complex Mixtures –

A Proof-Of-Concept Study using Crude Extracts of Chilean Mushrooms

Annegret Laub[1], Ann-Katrin Sendatzki[1], Götz Palfner[2], Ludger Wessjohann[1],

Jürgen Schmidt[1] and Norbert Arnold[1]

[1] Department of Bioorganic Chemistry, Leibniz Institute of Plant Biochemistry,

Weinberg 3, D-06120 Halle (Saale), Germany

[2] Departamento de Botanica, Facultad de Ciencias Naturales y Oceanograficas,

Universidad de Concepcion, Casilla 160-C, Concepcion, Chile

Supporting Information

Table S1. Origin of fungal material.

Species	Koll.	Date	Place	Leg./det.[1]
C. (D.) *austronanceiensis*	Chile 34/12	05.05.2012	Conguillío National Park	Arnold, Palfner
C. (D.) *icterina*	Chile 24/12	05.05.2012	Conguillío National Park	Arnold, Palfner
C. (D.) *icterinula*	Chile 3/12	27.04.2012	Curacautin, Laguna Blanca	Arnold, Palfner
C. (D.) *obscuro-olivea*	Chile 37/14	June 2014	Curacautin, Laguna Blanca	Arnold, Palfner
C. (D.) spec.	Chile 32/12	05.05.2012	Conguillío National Park	Arnold
C. (D.) *viridulifolius*	Chile 44/11	June 2011	Quillon, Cayumanque	Arnold

[1] Leg. = (lat.) legit, det. = (lat.) determinavit.

Table S2. Detected anthraquinones (1-6), their elemental composition and exact masses.

Compound	Elemental composition	$[M-H]^-$ Theoretical mass	$[M-H]^-$ Experimental mass (error) *C.* (D.) *austronanceiensis*	*C.* (D.) *icterina*	*C.* (D.) *icterinula*	*C. (D) obscuro- olivea*	*C.* (D.) spec.	*C.* (D.) *viridulifolius*
emodin (**1**)	$C_{15}H_9O_5^-$	269.0455	269.0450 (-2.0 ppm)	269.0451 (-0.8 ppm)	269.0448 (-2.6 ppm)	269.0450 (-1.9 ppm)	269.0449 (-2.2 ppm)	269.0453 (-0.8 ppm)
physicion (**2**)	$C_{16}H_{11}O_5^-$	283.0612	283.0611 (-0.5 ppm)	283.0610 (-0.8 ppm)	n.d.	283.0615 (-0.7 ppm)	283.0605 (-2.6 ppm)	283.0610 (-0.6 ppm)
endocrocin (**3**)	$C_{16}H_9O_7^-$	313.0354	313.0349 (-1.5 ppm)	313.0353 (-0.3 ppm)	313.0351 (-1.0 ppm)	313.0348 (-1.7 ppm)	n.d.	313.0351 (-1.0 ppm)
dermolutein (**4**)	$C_{17}H_{11}O_7^-$	327.0510	327.0505 (-1.7 ppm)	327.0505 (-1.6 ppm)	327.0503 (-2.1 ppm)	327.0506 (-1.4 ppm)	327.0501 (-2.9 ppm)	327.0507 (-1.1 ppm)
hypericin (**5**)	$C_{30}H_{15}O_8^-$	503.0772	503.0763 (-1.8 ppm)	n.d.	n.d.	503.0766 (-1.3 ppm)	n.d.	503.0767 (-1.1 ppm)
skyrin (**6**)	$C_{30}H_{17}O_{10}^-$	537.0827	537.0817 (-1.8 ppm)	n.d.	n.d.	537.0819 (-1.6 ppm)	n.d.	537.0822 (-1.0 ppm)

Table S3. Key ions in the negative ion ESI-MS^n spectra of skyrin (6).

Compound	Method	Scan Mode [*m/z*]	*m/z* [relative Intensity (%)]
skyrin (6) (fungal extract)	DESI	MS^2 (50%) *m/z* 537	493.0923 ($[M-H-CO_2]^-$, **100**), 469.0926 ($[M-H-C_3O_2]^-$, 80)
skyrin (6) (authentic reference compound)		MS^2 (35%) *m/z* 537	520.0783 ($[M-H-OH]^{\cdot -}$,), 519.0716 ($[M-H-H_2O]^-$), 509.0861 ($[M-H-CO]^-$), 493.0920 ($[M-H-CO_2]^-$), 475.0809 ($[M-H-H_2O-CO_2]^-$), 469.0922 ($[M-H-C_3O_2]^-$), 465.0956 $[M-H-CO-CO_2]^-$, 449.1021 ($[M-H-{}_2CO_2]^-$)
		MS^3 (35%) *m/z* 537-493	493.0908 ($[M-H- M-H-CO]^-$, 64), 475.0805 ($[M-H-H_2O-CO_2]^-$, 46), 465.0963 ($[M-H-CO-CO_2]^-$, **100**), 449.1014 ($[M-H-2CO_2]^-$, 97)
skyrin (6) (authentic reference compound)	direct infusion	MS^2 (30%) *m/z* 537	537.0829 ($[M-H]^-$, 96), 520.0801 ($[M-H-OH]^{\cdot -}$, 16), 519.0723 ($[M-H-H_2O]^-$, 19), 509.0879 ($[M-H-CO]^-$, 11), 493.0930 ($[M-H-CO_2]^-$, **100**), 475.0824 ($[M-H-H_2O-CO_2]^-$, 7), 469.0931 ($[M-H-C_3O_2]^-$, 72), 465.0981 ($[M-H-CO-CO_2]^-$, 8), 449.1033 ($[M-H-2CO_2]^-$, 14)
		MS^3 (40%) *m/z* 537-493	493.0930 ($[M-H-CO_2]^-$, 34), 475.0824 ($[M-H-H_2O-CO_2]^-$, 61), 465.0980 ($[M-H-CO-CO_2]^-$, 86), 449.1032 ($[M-H-2CO_2]^-$, **100**), 421.1083 ($[M-H-2CO_2-CO]^-$, 8)

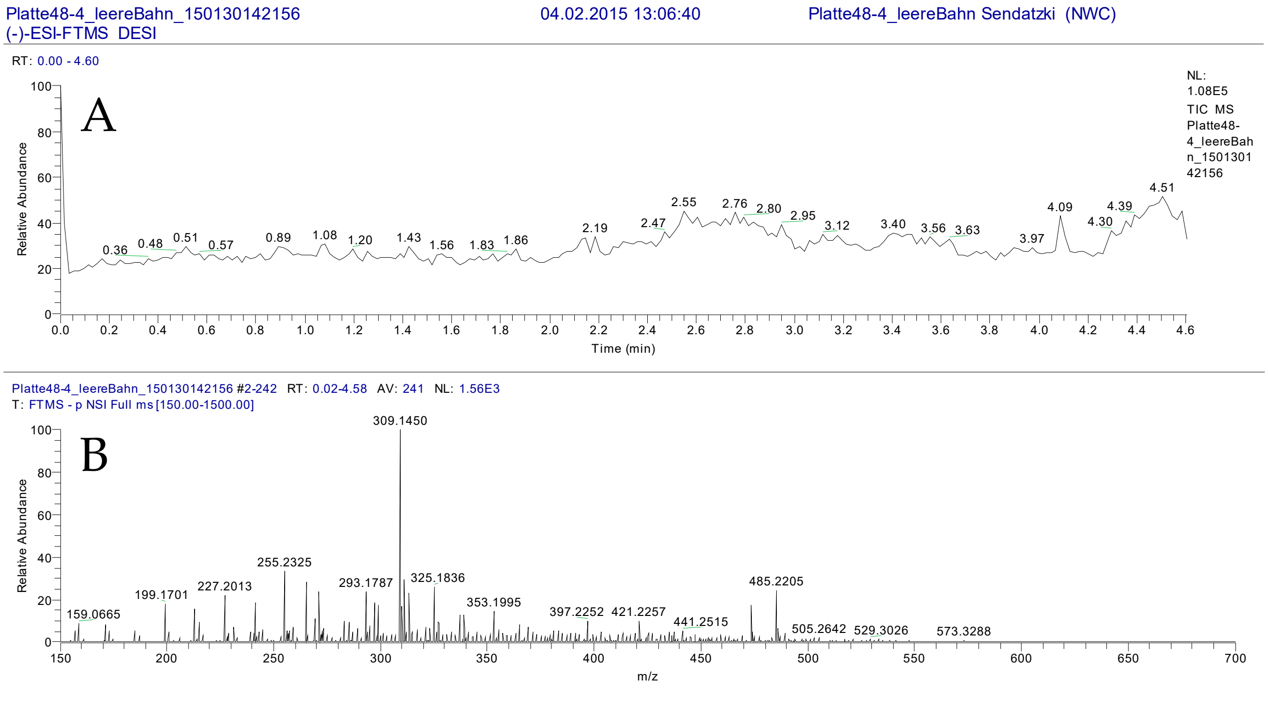

Figure S1: A) Total ion chromatogram of an empty HPTLC band after development with eluent system (toluene: ethyl formate: formic acid (10:5:3; *v/v/v*)) , B) Corresponding Full MS spectrum to A (averaged over Rt 0 - 4.6 min) showing background related peaks.

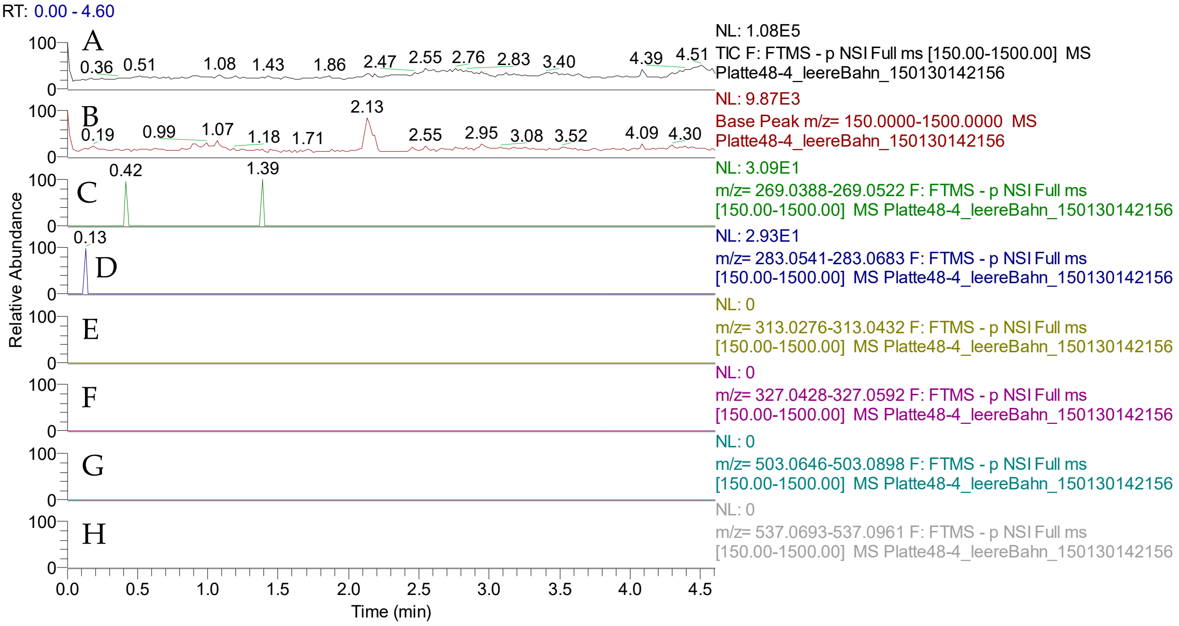

Figure S2. Total ion (A), base peak (B) and extracted ion chromatograms of an unspotted HPTLC band showing no anthraquinone related peaks (C - H).

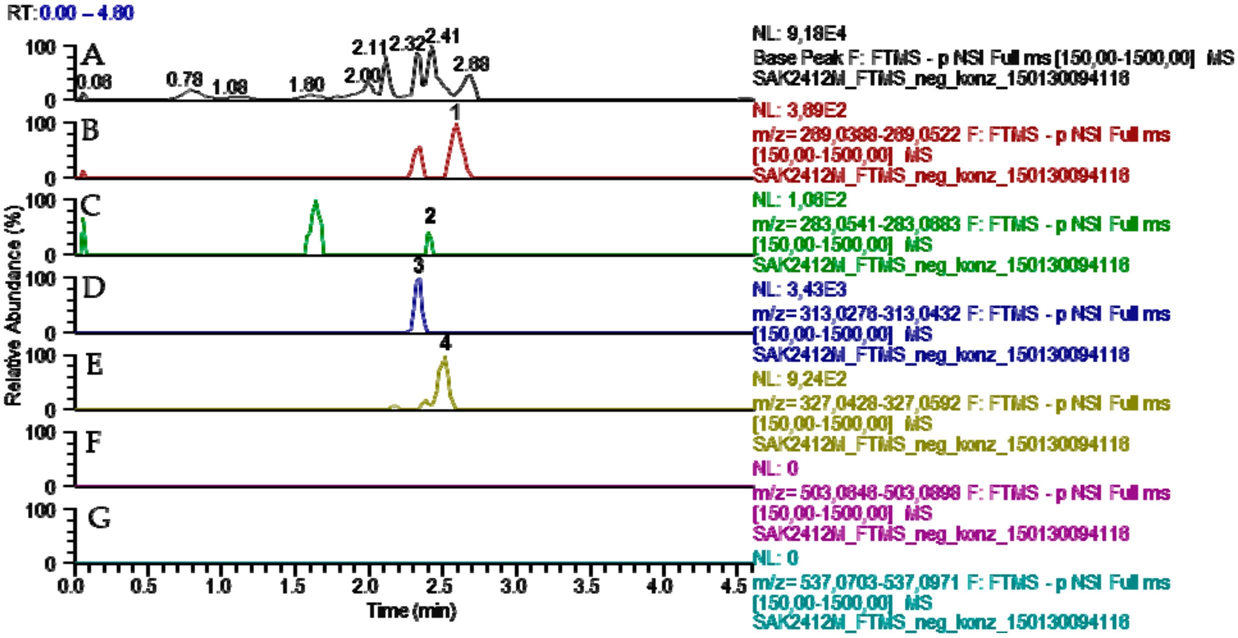

Figure S3. Base peak chromatogram (A) and extracted ion chromatograms (EICs, B - G) based on the theoretical masses of the investigated anthraquinones (1-6) obtained from the methanolic crude extract of *Cortinarius* (D.) *icterina.*

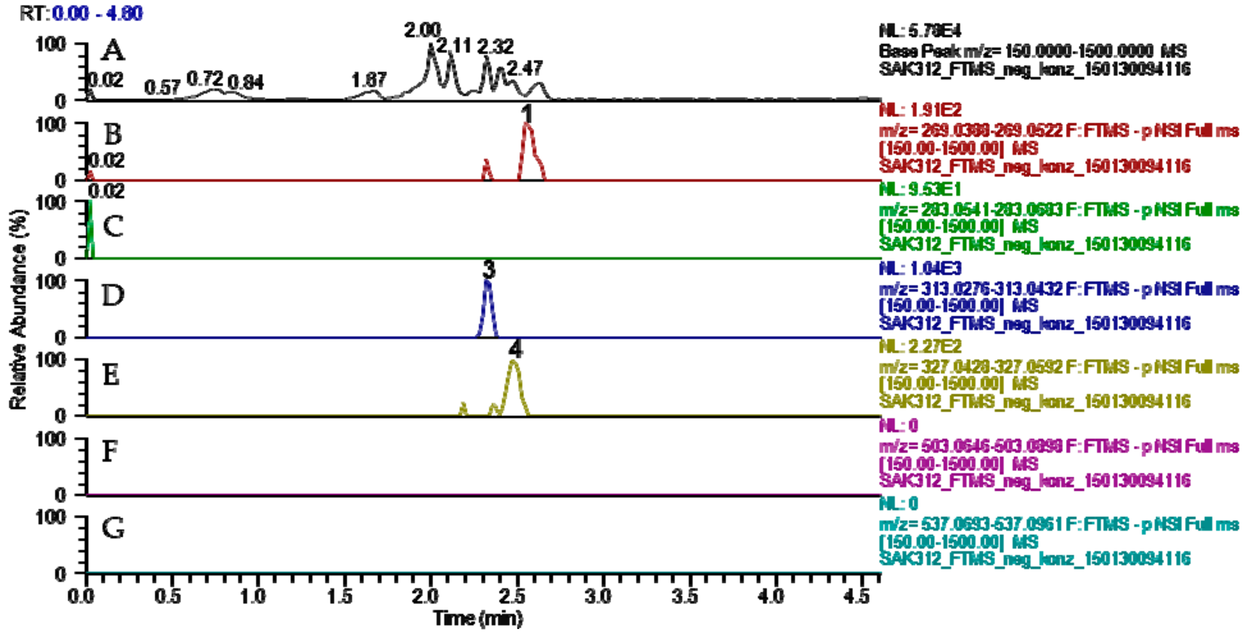

Figure S4. Base peak chromatogram (A) and extracted ion chromatograms (EICs, B - G) based on the theoretical masses of the investigated anthraquinones (1-6) obtained from the methanolic crude extract of *Cortinarius* (D.) *icterinula.*

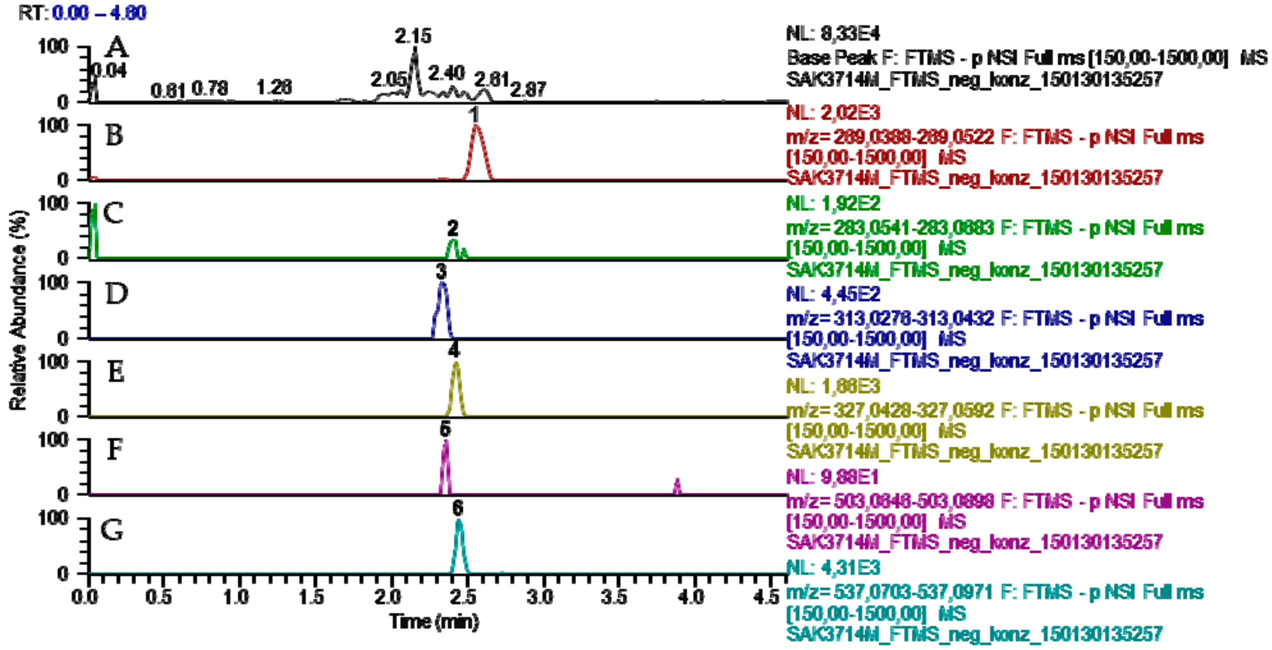

Figure S5. Base peak chromatogram (A) and extracted ion chromatograms (EICs, B - G) based on the theoretical masses of the investigated anthraquinones (1-6) obtained from the methanolic crude extract of *Cortinarius* (D.) *obscuro-olivea.*

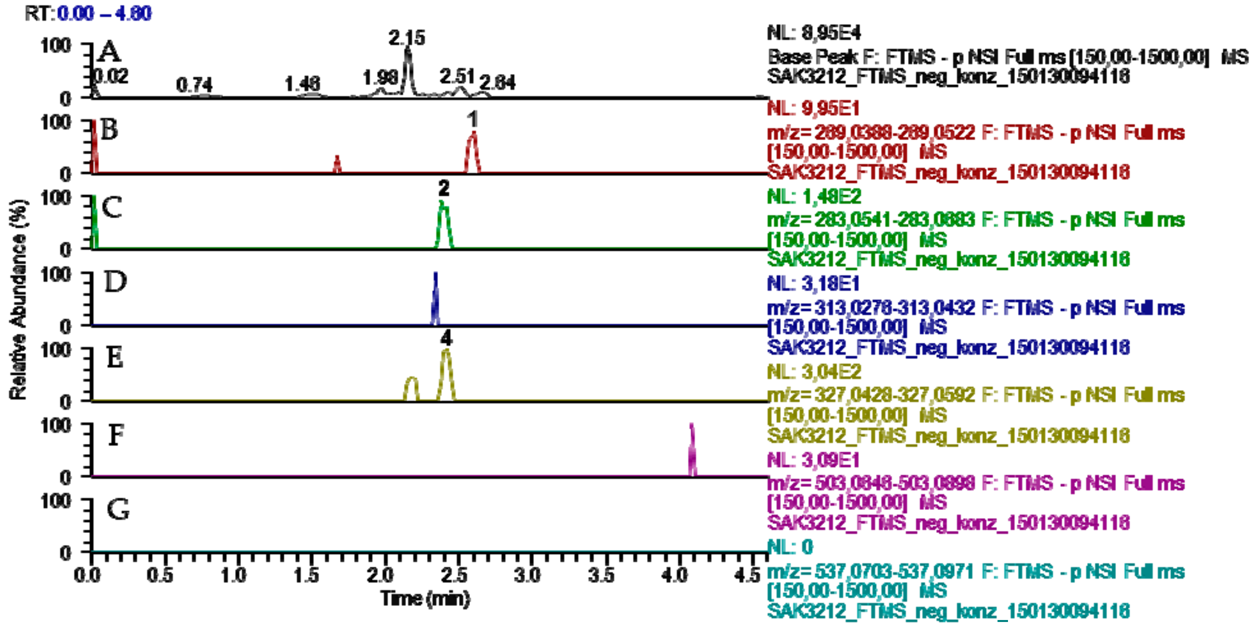

Figure S6. Base peak chromatogram (A) and extracted ion chromatograms (EICs, B - G) based on the theoretical masses of the investigated anthraquinones (1-6) obtained from the methanolic crude extract of *Cortinarius* (D.) spec.

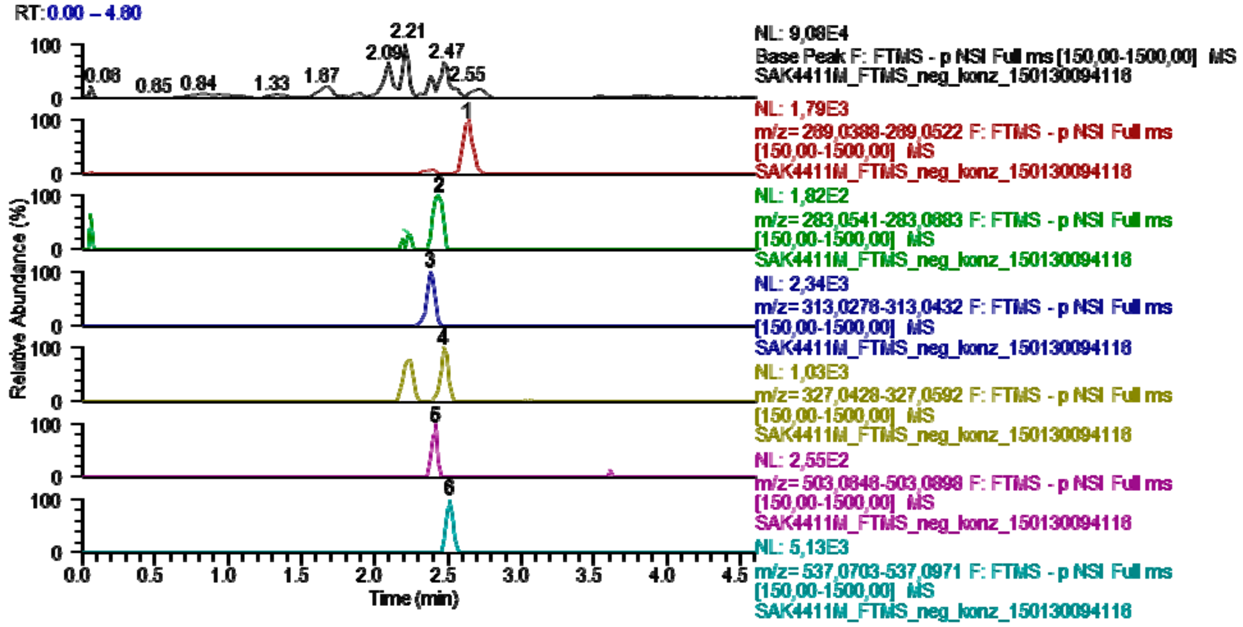

Figure S7. Base peak chromatogram (A) and extracted ion chromatograms (EICs, B - G) based on the theoretical masses of the investigated anthraquinones (1-6) obtained from the methanolic crude extract of of *Cortinarius* (D.) *viridulifolius.*

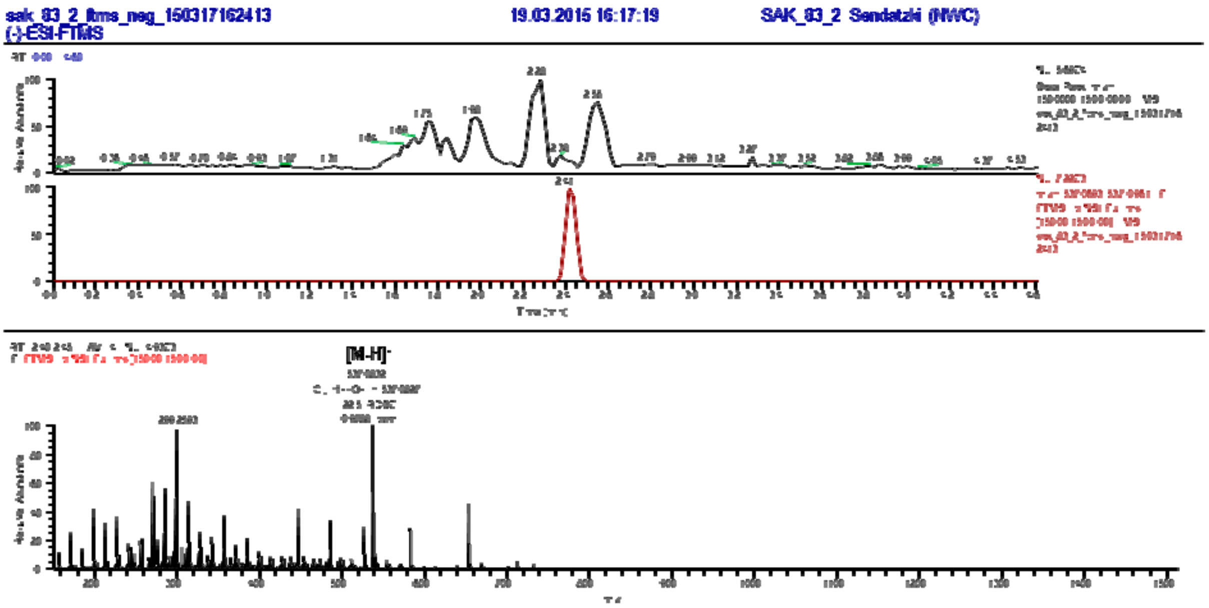

Figure S8. Total ion and extracted ion chromatogram (EIC) of reference compound skyrin (6) and the corresponding HRMS spectrum.

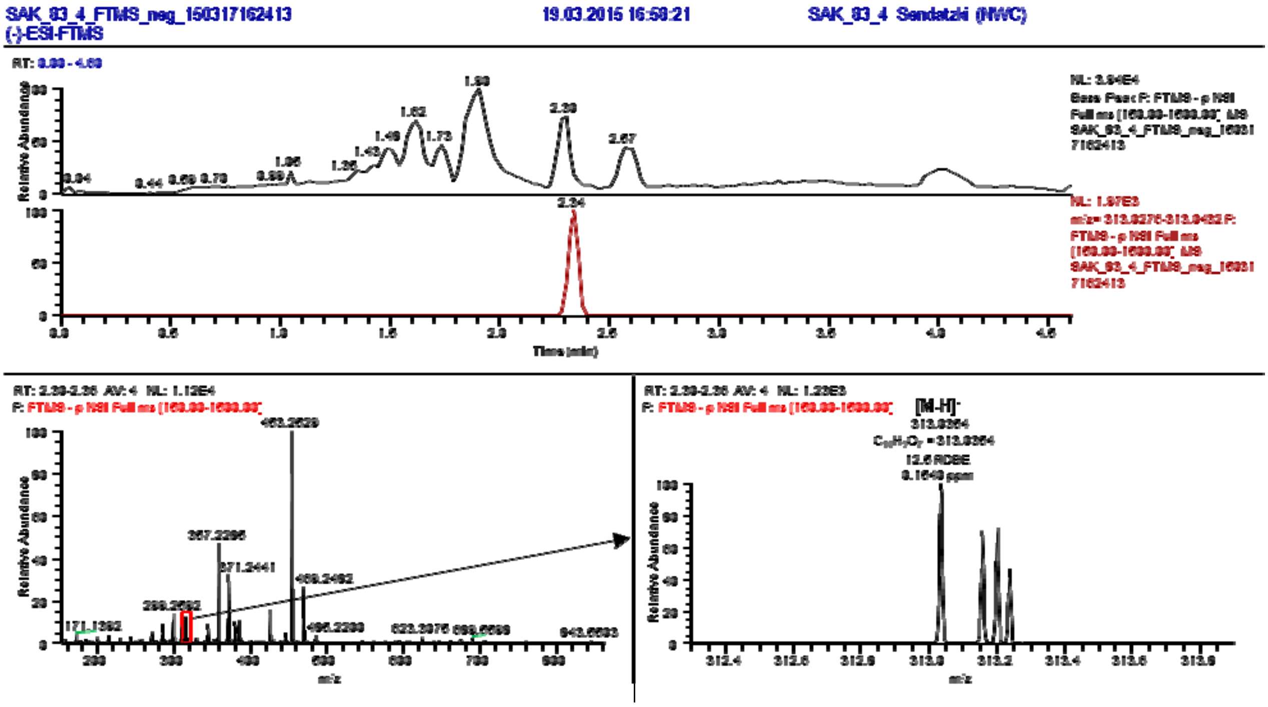

Figure S9. Total ion and extracted ion chromatogram EIC) of reference compound endocrocin (3) and the corresponding HRMS spectrum.

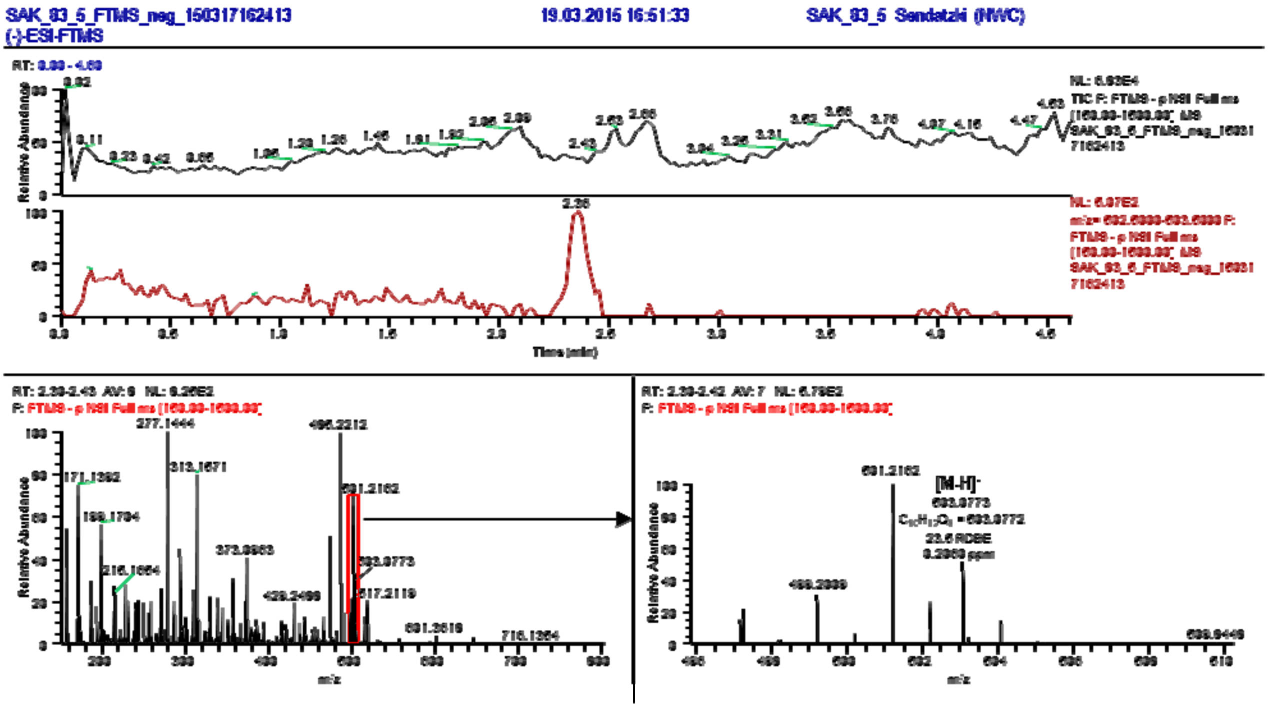

Figure S10. Total ion and extracted ion chromatogram (EIC) of reference compound hypericin (5) and the corresponding HRMS spectrum.

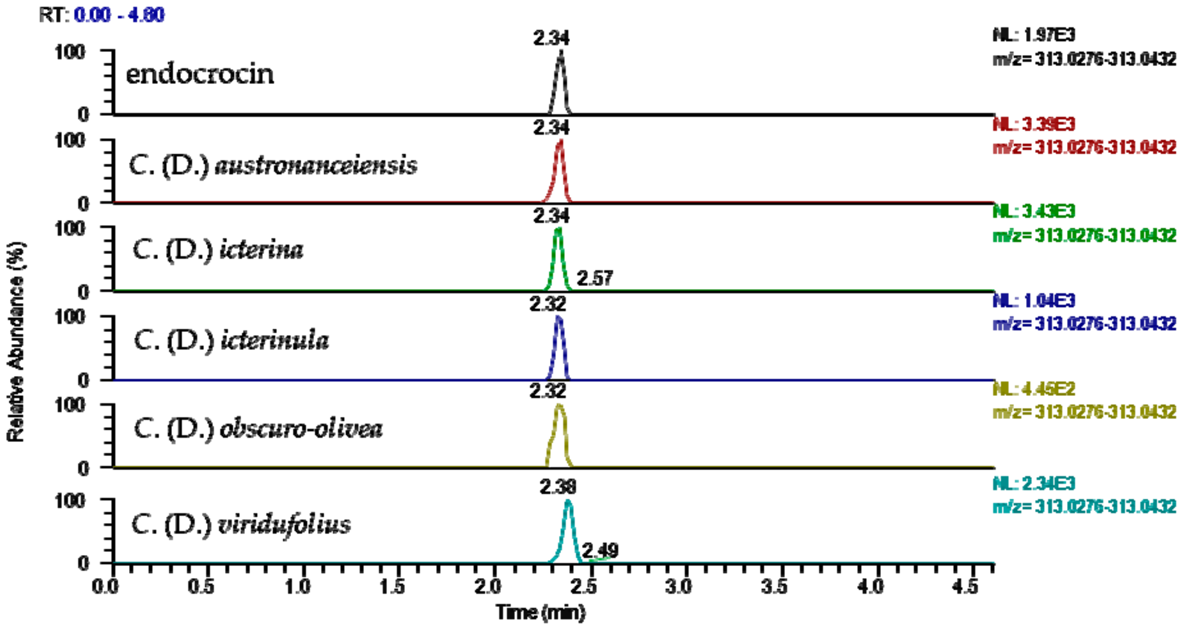

Figure S11. Comparison of extracted ion chromatograms (EICs) of endocrocin (3, *m*/*z* 313) acquired during DESI-HR-MS measurement of methanolic extract from *C.* (D.) species and the reference compound.
